# Subclinical Cardiac Remodeling in Borderline Hypertension: The Role of Advanced Imaging

**DOI:** 10.7759/cureus.112935

**Published:** 2026-07-18

**Authors:** Tamar Gaprindashvili, Rusudan Agladze, Inga Bochorishvili, Zurab Pagava

**Affiliations:** 1 Cardiology, Bokhua Memorial Cardiovascular Center, Tbilisi, GEO

**Keywords:** borderline hypertension, cardiac magnetic resonance, global longitudinal strain, left ventricular hypertrophy, myocardial fibrosis, speckle-tracking echocardiography, subclinical cardiac remodeling, t1 mapping

## Abstract

Among modifiable risk factors for cardiovascular diseases, hypertension is the most common. The most common form of cardiac damage in hypertensive patients is left ventricular hypertrophy (LVH), which is far from being the earliest and most sensitive marker of cardiac involvement. Microvascular dysfunction, impaired myocardial deformation, interstitial fibrosis, and neurohormonal activation are only some of the functional and structural elements that might not be detected by basic investigations such as electrocardiography and echocardiography, since they usually develop years before LVH. With this narrative review, we intend to highlight the role of advanced cardiac imaging in early, subclinical cardiac remodeling and (pre)hypertension. We delineated the concept of early cardiac remodeling, reviewed the limits of conventional LVH detection, and discussed the diagnostic and prognostic value of speckle-tracking echocardiography with global longitudinal strain (GLS) and of cardiac magnetic resonance with late gadolinium enhancement, T1 mapping, and extracellular volume (ECV) quantification. Left ventricular ejection fraction impairment occurs later than GLS decline, whereas T1 mapping and ECV allow for interstitial fibrosis detection at the tissue level. Novel strain methods, circulating biomarkers, and artificial intelligence are embraced as complementary tools for earlier detection and risk stratification. The review was based on a non-systematic search of the PubMed and Scopus databases for records through April 2026, ultimately including 56 references. The experience of advanced imaging techniques can identify subclinical myocardial alterations far earlier than conventional investigations; thus, they should be considered as potential complementary methods and not alternatives.

## Introduction and background

Hypertension remains among the most consequential modifiable risk factors for global mortality and cardiovascular disease. According to the World Health Organization, an estimated 1.4 billion adults aged 30-79 years were living with hypertension in 2024, corresponding to approximately 33% of that population [1]. This figure has more than doubled since 1990, with the greatest burden in low- and middle-income countries, where roughly two-thirds of affected adults now live [1,2]. Even where antihypertensive pharmacotherapy is broadly accessible, only about one in five treated adults achieves target blood pressure control [1,3]. These statistics highlight the need for the early detection of target organ damage, particularly hypertension-induced cardiac remodeling, before irreversible structural changes take place.

Hypertensive heart disease (HHD) is characterized by the development of left ventricular hypertrophy (LVH), a well-known independent risk factor for cardiovascular morbidity and mortality [4]. The current paradigm of hypertensive remodeling assumes that pressure overload is the predominant mediator of the transition from hypertrophy to heart failure (HF), via a compensatory phase of concentric hypertrophy aimed at normalizing wall stress and eventually leading to chamber dilation and HF [5]. But there is evidence that myocardial damage occurs well in advance of the development of echocardiographically evident LVH involving interstitial fibrosis, neurohormonal activation, microvascular ischemia, and subtle abnormalities of myocardial contractility [6]. This preclinical phase offers potential for therapeutic intervention to prevent progression of hypertensive myocardial disease.

Borderline hypertension (also called prehypertension or, as currently used in Europe, high-normal blood pressure) is characterized by blood pressure measurements higher than optimal but below the threshold for hypertension. According to the 2017 American College of Cardiology/American Heart Association (ACC/AHA) guideline, blood pressure values in the range of 120-129/<80 mmHg are considered elevated, and those in the range of 130-139/80-89 mmHg correspond to stage 1 hypertension [7]. The 2018 European Society of Cardiology/European Society of Hypertension (ESC/ESH) guideline set the diagnostic threshold for hypertension at 140/90 mmHg and classified blood pressure in the range of 130-139/85-89 mmHg as high-normal [8]. This threshold has since been reaffirmed by the 2023 ESH guideline [9] and by the 2024 ESC guideline for the management of elevated blood pressure and hypertension, which introduced an “elevated BP” category (120-139/70-89 mmHg) broadly comparable to the earlier high-normal/prehypertension framework [10]. Because terminology differs across guidelines, this review uses “borderline hypertension” as an umbrella term for blood pressure above optimal levels but below, or at the lower boundary of, diagnostic hypertension, encompassing the ACC/AHA elevated/stage 1 hypertension categories and the European high-normal/elevated BP categories. Patients with borderline hypertension have an increased risk of cardiovascular disease compared with normal patients, and many already have underlying cardiac changes that are undetectable by conventional means [11].

**Figure 1:**
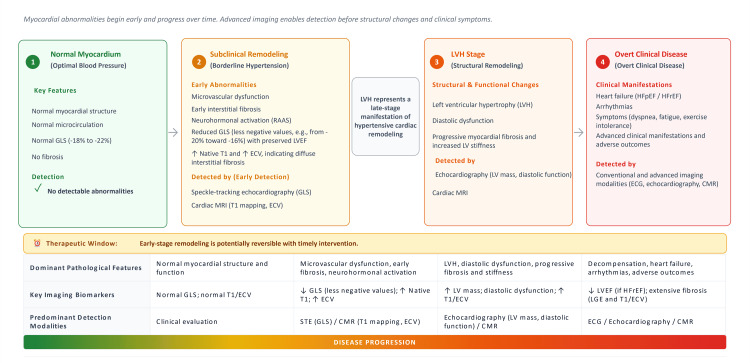
Timeline of hypertensive cardiac remodeling: from subclinical injury to overt disease GLS: global longitudinal strain; LVEF: left ventricular ejection fraction; RAAS: renin-angiotensin-aldosterone system; T1: native t1 mapping; ECV: extracellular volume; LGE: late gadolinium enhancement; CMR: cardiac magnetic resonance; ECG: electrocardiography; HFpEF: heart failure with preserved ejection fraction; HFrEF: heart failure with reduced ejection fraction Conceptual framework summarized from references [5,6] Figure created by the author (Tamar Gaprindashvili) using Microsoft PowerPoint.

Given the understanding that organ damage may precede clinically established disease, there is now an increasing focus on the identification of the earliest changes occurring in the myocardium. In this review, we discuss the pathophysiology of subclinical cardiac remodeling in borderline hypertension and the potential role of advanced imaging techniques such as speckle tracking echocardiography (STE) and cardiac magnetic resonance (CMR) in screening and risk assessment (Figure 1).

## Review

Search strategy

This narrative review was carried out and reported according to the quality criteria of SANRA (Scale for the Assessment of Narrative Review Articles) [12]. Two investigators searched the electronic databases PubMed/MEDLINE and Scopus for articles published in English between January 1990 and April 2026. The following keywords and/or Medical Subject Headings (MeSH) terms were used: (“Hypertension” OR “prehypertension” OR “borderline hypertension”) AND (“Hypertensive heart disease”) OR (“cardiac remodeling” OR “myocardial remodeling”) OR (“left ventricular hypertrophy”) OR (“myocardial fibrosis”) OR (“global longitudinal strain”) OR (“speckle-tracking echocardiography”) OR (“cardiac magnetic resonance”) OR (“T1 mapping”) OR (“extracellular volume”) OR (“cardiac biomarkers”) OR (“artificial intelligence”). Reference lists of selected articles were also screened manually. Original articles, consensus documents, guidelines, reviews, and meta-analyses, which were considered relevant to the research, were reviewed. Publications in languages other than English, abstracts from scientific sessions, and full-text not available were excluded. The search strategy is summarized in Table 1.

**Table 1:** Search strategy used for this narrative review MeSH: Medical Subject Headings; SANRA: Scale for the Assessment of Narrative Review Articles. Search strategy conducted in accordance with reference [12].

Search parameter	Details
Databases searched	PubMed/MEDLINE; Scopus
Time period	January 1990 to April 2026
Keywords/MeSH terms	(“Hypertension” OR “prehypertension” OR “borderline hypertension”) AND (“hypertensive heart disease” OR “cardiac remodeling” OR “myocardial remodeling” OR “left ventricular hypertrophy” OR “myocardial fibrosis” OR “global longitudinal strain” OR “speckle-tracking echocardiography” OR “cardiac magnetic resonance” OR “T1 mapping” OR “extracellular volume” OR “cardiac biomarkers” OR “artificial intelligence”)
Additional sources	Manual screening of reference lists from selected articles
Inclusion criteria	Original articles, consensus documents, clinical guidelines, reviews, and meta-analyses relevant to subclinical cardiac remodeling, hypertension, and advanced imaging, published in English
Exclusion criteria	Non-English publications; conference abstracts; articles without retrievable full text
Records identified/included	56 references included in the final narrative synthesis

Pathophysiology of early remodeling

In summary, this review indicates that in the development of hypertensive cardiac remodeling, hemodynamic and non-hemodynamic factors interact to influence changes in cardiac structure and function. Generally, these mechanisms operate in full force in hypertension and at a reduced scale in borderline hypertension, the latter determining the subtle changes that occur prior to LVH. It is the complex nature of these processes that warrants instruments of detection other than mere thickness of the ventricular wall to reveal early modifications.

Myocardial fibrosis

Myocardial fibrosis appears as one of the first histological hallmarks of hypertensive heart disease and is a key driver of the development of cardiac dysfunction. Collagen deposition occurs in the perivascular and interstitial space of the hypertensive myocardium, primarily due to activated fibroblasts and profibrotic factors such as transforming growth factor beta (TGF-β), connective tissue growth factor, and galectin-3 [6]. Myocardial fibrosis stiffens the heart, impairs ventricular relaxation, and leads to diastolic dysfunction, which is one of the most common early complications of hypertension [13].

Two types of fibrosis need to be distinguished because they differ in mechanism, reversibility, and imaging characteristics. Reactive interstitial fibrosis is an early diffuse expansion of the interstitial and perivascular space that is at least partially reversible and is not associated with cardiomyocyte loss. It is not detectable by conventional imaging or late gadolinium enhancement (LGE) methods but is detectable by native T1 mapping and extracellular volume (ECV) measurement. Reparative (replacement) fibrosis develops later as focal scarring in regions of cardiomyocyte loss. This type of fibrosis is the typical substrate detected by LGE. Diffuse interstitial fibrosis may be found in hypertensive patients without established LVH and therefore is undetectable by techniques that require marked structural alteration [14]. Reactive collagen production early on resists ventricular remodeling and is cardioprotective. Later, it becomes pathogenic and excessive fibrosis impairs diastolic as well as systolic function [5].

Neurohormonal activation

The renin-angiotensin-aldosterone system (RAAS) and the sympathetic nervous system play important roles in hypertrophic cardiac remodeling [15]. Through the angiotensin II type 1 receptor (AT1R), which is expressed by both cardiomyocytes and cardiac fibroblasts, angiotensin II induces cardiomyocyte hypertrophy, fibroblast proliferation, and collagen production. Via mineralocorticoid receptors expressed by cardiac fibroblasts, aldosterone promotes cardiac fibrosis that is independent of its ability to raise blood pressure. Excess catecholamines induce cardiomyocyte apoptosis and adverse remodeling via beta-adrenergic stimulation.

The classical angiotensin-converting enzyme (ACE)/angiotensin II/AT1R arm has a counterbalance constituted by the angiotensin-converting enzyme 2 (ACE2), angiotensin-(1-7), and the Mas receptor [16,17]. The angiotensin-(1-7), acting on the Mas receptor, has vasodilatory, antifibrotic, anti-hypertrophic, and anti-inflammatory actions, while an unbalanced activation of the classical arm would lead to fibrosis and remodeling [17,18]. These systems are commonly activated during the phase at which the hemodynamic overload alone would be sufficient to promote structural alterations, thus, patients with borderline hypertension could reveal neurohormonally-driven modifications [19].

Microvascular dysfunction

Coronary microvascular dysfunction plays a critical role in the initial phase of hypertensive cardiac damage. Coronary microcirculation exhibits several abnormalities such as thickening of arteriolar walls, perivascular fibrosis, and rarefaction of capillaries, which cumulatively restrict the coronary flow reserve (CFR) progressively [20]. These changes in conjunction with endothelial dysfunction cause repetitive ischemia in the subendocardial region and induce fibrosis and damage to cardiomyocytes. Decreased CFR has been linked with left ventricular (LV) systolic and diastolic dysfunction in HHD [21]. Plus, microvascular ischemia and fibrosis have been related to the genesis of ventricular tachyarrhythmias and sudden cardiac death in hypertensive patients [22,23]. It is important to note that abnormalities in the microcirculation have been shown to precede and contribute to LVH, further substantiating the concept of organ damage before the onset of clinical symptoms. The pathophysiological mechanisms discussed above and their corresponding imaging findings are summarized in Table 2.

**Table 2:** Pathophysiological mechanisms and corresponding imaging findings RAAS: renin-angiotensin-aldosterone system; GLS: global longitudinal strain; STE: speckle-tracking echocardiography; ECV: extracellular volume; CMR: cardiac magnetic resonance; T1: native T1 mapping; LV: left ventricle; LVEF: left ventricular ejection fraction. Mechanisms and imaging correlates summarized from references [6,13,14,19-25].

Pathophysiological mechanism	Structural/functional alteration	Imaging marker(s)	Clinical implication
Interstitial fibrosis	Increased myocardial stiffness; impaired ventricular relaxation	Increased native T1; increased extracellular volume (ECV) on CMR	Early diastolic dysfunction; progression toward heart failure
Microvascular dysfunction	Reduced coronary flow reserve; subendocardial ischemia	Reduced global longitudinal strain (GLS) on STE	Early subclinical systolic dysfunction
Neurohormonal activation (RAAS, sympathetic nervous system)	Cardiomyocyte hypertrophy; fibroblast activation; collagen deposition	Increased left ventricular mass (echocardiography/CMR); reduced GLS	Structural remodeling and progression of hypertensive heart disease
Subendocardial injury	Selective impairment of longitudinal myocardial fibers	Reduced GLS with preserved LVEF	Earliest detectable functional abnormality
Progressive myocardial remodeling	Left ventricular hypertrophy; increased fibrosis; reduced compliance	Increased LV mass; diastolic dysfunction; elevated T1/ECV	Increased cardiovascular risk; transition to overt disease

Limitations of conventional LVH detection

The diagnosis of LVH has traditionally relied on electrocardiography (ECG) and transthoracic echocardiography. Both remain accessible and clinically relevant, but both have considerable shortcomings for detecting subclinical myocardial damage.

Electrocardiographic Limitations

The diagnostic criteria for LVH by ECG, including the widely used Sokolow-Lyon criteria and Cornell voltage criteria, all have high specificity and low sensitivity. In the analysis of 4684 subjects from the Framingham Heart Study, the overall sensitivity of ECG in detecting LVH was 6.9% with an overall specificity of 98.8% [26]. The vast majority of patients with anatomical LVH will be categorized as normal. Sensitivity of the ECG criteria for anatomical LVH varies according to sex, body habitus, age, and ethnicity with obesity, female sex, and advanced age all impacting the accuracy [27]. QRS voltage amplitude is dependent not only on myocardial mass but also on its distance from the electrodes, thickness of the chest wall, myocardial fibrosis, and conduction delays [27]. Novel criteria have been developed, including the Peguero-Lo Presti criteria, which improve sensitivity and continue to improve upon the limitations of early and subtle cardiac remodeling [28]. The ECG provides no insight into diastolic function, fibrosis, or subclinical systolic dysfunction. Despite these limitations, the ECG continues to be a critical tool for population screening and risk evaluation in established cardiovascular risk scores, and regression of ECG-LVH with treatment confers a better prognosis.

Echocardiographic Limitations

Two-dimensional echocardiography is widely used in the clinical evaluation of LV mass and geometry due to its availability, speed, and non-invasiveness. However, it is operator-dependent, subject to significant inter-observer variability, and its accuracy is limited by its geometric assumptions [29]. Compared to CMR, echocardiography overestimates the presence of LVH in 15% of subjects and underestimates it in 14% of subjects [30]. Linear dimensions used in the Devereux formula to calculate left ventricular mass (LVM) = 0.8 × [1.04 × (LVEDD + PWT + IVS)3 − LVEDD3] + 0.6 g, where LVEDD is LV end-diastolic diameter, PWT is posterior wall thickness, and IVS is interventricular septum thickness, and assumes the LV to be a prolate-ellipsoid, which may not be true in asymmetric hypertrophy. Conventional metrics like left ventricular ejection fraction (LVEF) are insensitive to subtle alterations in myocardial contractility; LVEF may remain normal even in the face of significant subendocardial dysfunction because of compensatory circumferential and radial strains that contribute to maintaining pump function even when longitudinal strain is severely compromised [24]. The relative capabilities of the two modalities are summarized in Table 3.

**Table 3:** Comparison of imaging modalities for the detection of hypertensive cardiac remodeling ECG: electrocardiography; LVH: left ventricular hypertrophy; LV: left ventricle; GLS: global longitudinal strain; STE: speckle-tracking echocardiography; LVEF: left ventricular ejection fraction; CMR: cardiac magnetic resonance; LGE: late gadolinium enhancement; T1: native T1 mapping; ECV: extracellular volume. Modality characteristics summarized from references [26,27,29-35]

Imaging modality	Key parameters assessed/what it detects	Strengths	Limitations	Stage of disease detected
Electrocardiography (ECG)	Electrical activity and voltage criteria for LVH; electrical abnormalities (LVH detection has low sensitivity)	Widely available; low cost; rapid assessment	Very low sensitivity for early disease; cannot assess myocardial structure/function or detect subclinical remodeling or fibrosis	Late (advanced structural changes)
Conventional echocardiography	LV mass and geometry; systolic and diastolic function; structural heart changes	Widely available; non-invasive; provides comprehensive structural and functional information	Operator-dependent; relies on geometric assumptions; limited sensitivity for early myocardial dysfunction	Intermediate (structural remodeling stage)
Speckle-tracking echocardiography (STE; GLS)	GLS and myocardial deformation; subclinical systolic dysfunction with reduced GLS and preserved LVEF	Sensitive for subclinical systolic dysfunction; angle-independent; detects changes before LVEF decline	Requires good image quality and expertise; vendor variability	Early (subclinical stage)
Cardiac magnetic resonance – LGE	Focal (replacement) myocardial fibrosis; late gadolinium enhancement	High spatial resolution; high accuracy for focal myocardial fibrosis	Limited ability to detect diffuse interstitial fibrosis; requires a contrast agent	Intermediate to late (focal fibrosis stage)
Cardiac magnetic resonance – T1 mapping/ECV	Diffuse interstitial fibrosis; native T1 and extracellular volume; quantitative tissue characterization	Most advanced non-invasive method for diffuse interstitial fibrosis; quantitative and reproducible	Higher cost; more limited availability; longer acquisition and post-processing time; contrast required for ECV (not for native T1)	Early (subclinical stage)

Role of speckle-tracking echocardiography

Recently, a promising technique known as STE has been developed to assess myocardial function. This methodology follows the movement of unique acoustic patterns ("speckles") in the myocardium during contraction and relaxation, allowing for the quantification of myocardial deformation in longitudinal, circumferential, and radial directions without angle dependency [31].

Global longitudinal strain in hypertension

Global longitudinal strain (GLS) represents the percentage of myocardial deformation (shortening) in the longitudinal direction during systole and is the most clinically relevant STE-based measurement. GLS has been shown to detect and predict cardiovascular disease better than LVEF in several cardiac conditions. It is being increasingly utilized in the clinical setting because of its sensitivity to early LV dysfunction [25]. In general, a strain value between −18% and −22% is considered normal, and less negative values indicate impaired deformation. Values less negative than the cutoff value of approximately −16% are generally considered abnormal, and values between −16% and −18% are thought to be in the "grey zone." GLS has been found to decrease in hypertensive individuals even when LVEF is normal, with a greater decrease observed in patients with heart failure with preserved ejection fraction [36]. Because the longitudinally oriented fibers of the subendocardium are vulnerable to pressure overload and ischemia, the longitudinal function is the first to deteriorate in HHD [37]. Thus, GLS may be used in the hypertensive population to detect subclinical systolic dysfunction when other parameters remain normal.

It should be noted that GLS values are not comparable across systems. The European Association of Cardiovascular Imaging (EACVI)/American Society of Echocardiography (ASE)/Industry GLS, strain software comparison study showed that modest but statistically significant differences in GLS values exist between vendor software due to differences in the speckle tracking algorithms [32]. Therefore, follow-up studies should be performed on the same platform whenever possible. The EACVI/ASE/Industry standardization study is making progress in reducing these differences.

Subclinical dysfunction and compensatory mechanisms

The ability of STE to detect subclinical systolic dysfunction is important because it provides us with insight into the mechanics of the hypertensive heart. As the longitudinal function becomes increasingly compromised, the short-axis function (circumferential and radial) is preserved or even enhanced to compensate for the loss of longitudinal function, thereby preserving cardiac output and ejection fraction [24]. It is important to detect the abnormalities during this stage of the disease as the disease may be reversible at this stage [25]. Antihypertensive treatment has the potential to reverse, at least in part, the impairment of GLS, especially in those who have the greatest degree of impairment at baseline, suggesting the presence of a window of opportunity to treat the disease during the subclinical phase [38].

Prognostic value of GLS

A lower GLS independently predicted incident HF, myocardial infarction, and cardiovascular mortality in a community-based sample after adjusting for age, sex, heart rate, hypertension, systolic blood pressure, and LVEF, with median follow-up of 11 years [39]. Plus, GLS predicted outcomes in all subgroups, suggesting that GLS provides prognostic information beyond traditional risk models. GLS is an independent predictor of cardiovascular events in patients with hypertension and hypertensive cardiomyopathy [24]. The EACVI/ASE/Industry consensus document on deformation imaging recommends GLS as a reliable and clinically validated measure of LV systolic function [31]. STE represents an appealing alternative in population screening because of its relatively low cost and wide availability [40].

Role of cardiac magnetic resonance imaging

Cardiac magnetic resonance imaging (CMR) is considered the gold standard for evaluation of cardiac morphology and function. In HHD, CMR provides unique insight into myocardial tissue composition that is unmatched by other non-invasive techniques [13].

Late gadolinium enhancement

LGE allows for the visualization of focal (replacement) fibrosis and scar. Following intravenous administration of gadolinium-based contrast agents, focal fibrotic myocardium appears hyperintense in T1-weighted imaging due to delayed wash-out of contrast relative to normal myocardium, as a result of increased extracellular space from increased collagen and decreased cardiomyocytes. LGE is capable of detecting mid-wall fibrosis, most commonly seen in the interventricular septum, which carries a poor prognosis and is associated with an increased risk of ventricular arrhythmia, hospitalization for HF, and death [33]. LGE detects differences in contrast distribution between abnormal and normal myocardium, and absolute quantification of interstitial (diffuse) fibrosis, representative of the entire myocardium, is not possible [34]. Early stages in HHD consist primarily of diffuse interstitial fibrosis rather than replacement fibrosis, and therefore the earliest myocardial injuries cannot be detected with LGE alone. It should be noted that both LGE and extracellular volume fraction (ECV) imaging methods require administration of gadolinium-based contrast agents, necessitating discussion of risk and screening of renal function, and contraindications such as pregnancy.

T1 mapping and extracellular volume quantification

T1 mapping represents a new technique in tissue characterization, where T1 relaxation times are measured, reflecting the underlying tissue characteristics of the myocardium. While LGE imaging provides a qualitative assessment of focal scar, T1 mapping provides a quantitative assessment of diffuse myocardial disease that characterizes even the early interstitial fibrosis of hypertensive remodeling [35]. Native T1, measured prior to contrast administration, is elevated in conditions where there is excess water or fibrosis in the extracellular space, and pre- and post-contrast T1 measurements, when used in conjunction with hematocrit of the blood, provide a measure of the ECV [41].

Native T1 and ECV detect subtle LV fibrosis in primary hypertension even in the absence of LGE [14]. Native T1 and ECV increased with the duration of hypertension in a hypertensive animal model, and both were significantly associated with collagen volume fraction on histological examination, demonstrating that native T1 and ECV are tissue-level fibrosis measures [42]; interstitial fibrosis preceded the decline in LVEF, establishing that T1 mapping detects abnormalities at the subclinical stage of disease [43]. A recent systematic review and meta-analysis has demonstrated significant increases in both T1 and ECV in populations prior to the development of heart failure compared to controls [44].

Native T1 mapping does not require contrast, while ECV quantification requires gadolinium administration. The ability of native T1 to detect diffuse fibrosis without the need for a contrast agent makes it an attractive option for serial studies or population screening, but native T1 is far more sensitive to variations in pulse sequence, magnetic field strength and vendor, and less specific since non-fibrotic pathologies such as edema, infiltration, and amyloid can also elevate native T1. ECV is more reproducible across sites and easier to relate to underlying biology, but requires a contrast agent, which has added cost and contraindications. Other emerging contrast-free techniques such as T2 mapping and T1ρ may decrease CMR dependence on gadolinium further but have not yet been validated in a clinical setting for serial hypertensive heart disease screening.

Comprehensive cardiac assessment

In addition to tissue characterization, CMR imaging provides accurate and reproducible assessment of ventricular volumes, ventricular mass, and global ventricular function without the need for geometric assumptions used in echocardiographic calculations. The LV mass derived from CMR is considered the reference standard, and CMR imaging has been shown to have significantly lower inter-observer variability compared with echocardiography [29]. CMR imaging feature-tracking strain has been shown to provide similar myocardial strain imaging capability as STE, offering the opportunity to link functional abnormalities with tissue-level pathology in one test, which makes CMR an ideal tool to comprehensively characterize hypertensive cardiomyopathy [29].

Clinical implications

Earlier Diagnosis

In this regard, the diagnostic capacity of the most recent imaging modalities to identify subclinical remodeling carries major implications for the clinical management of hypertensive patients. The ability to identify myocardial damage in a potentially reversible phase may allow targeted interventions to prevent permanent structural alterations. Advanced imaging techniques may identify a subgroup of patients posing high-risk features among the large population of subjects with borderline or mildly elevated blood pressure, who may have the greatest benefit from early pharmacologic treatments. These observations are in line with the recommendations of the American Heart Association and the latest European Society of Cardiology guidelines emphasizing the importance of early detection of hypertension-mediated organ damage [7,10].

Risk Stratification

Improved cardiovascular risk prediction algorithms using imaging modalities can be achieved by incorporating subclinical LV structure and function abnormalities from echocardiography. Specifically, community-based studies show that any LV abnormalities at baseline echocardiography improve the predictive ability of the 2013 ACC/AHA risk score (Pooled Cohort Equations) by a significant extent [45]. The presence of any baseline LV abnormalities in individuals at moderate-high risk of atherosclerotic cardiovascular disease conferred a three-fold increased risk for cardiovascular events. On top of that, CMR-derived measures of ECV and GLS also predict incident HF and cardiovascular death beyond traditional risk factors and cardiovascular risk scores [39].

Treatment Timing and Monitoring

Additional imaging parameters such as GLS and ECV can be utilized for monitoring antihypertensive therapy and for steering treatment. Subclinical systolic and diastolic dysfunction identified on STE is partly reversible with adequate control of blood pressure, especially in those with worse dysfunction at baseline [38], reflecting the concept of a reversibility window for treatment. Drugs like angiotensin-converting enzyme blockers and angiotensin receptor blockers can be particularly effective in reversing ventricular remodeling due to their specific action on RAAS and are first-line therapeutic agents in the setting of HHD [8].

Future directions

Biomarker-Imaging Integration

Emerging cardiac biomarkers in circulating blood are proving useful adjuncts to imaging for detection and risk stratification of HHD. High-sensitivity cardiac troponin and natriuretic peptides (B-type natriuretic peptide (BNP) and N-terminal pro-B-type natriuretic peptide (NT-proBNP)) are indicators of myocardial stress and injury and are strongly associated with systolic blood pressure, LV mass, diastolic function, and myocardial fibrosis in hypertensive cohorts [46]. A recent meta-analysis comprising 17 studies and over 70,000 individuals without known HF showed that higher levels of BNP/NT-proBNP and troponin were associated with increased risk for major adverse cardiovascular events in hypertensive patients [47]. The combination of circulating cardiac biomarkers and advanced cardiac imaging techniques may provide a multimodal and comprehensive risk assessment tool that provides a unique yet complementary insight into myocardial injury, hemodynamic stress, and anatomic remodeling. Interestingly, both of these biomarkers decrease in response to aggressive antihypertensive therapy [48].

Artificial Intelligence in Cardiac Imaging

Artificial intelligence (AI) and machine learning are making tremendous advances in cardiac imaging. Convolutional neural networks have been shown to perform automated image analysis, pattern recognition, and diagnostic classification at levels comparable to human experts for select applications [49]. In echocardiography, AI has been used for automated strain analysis, detection of LVH, classification of cardiomyopathies, and detection of subclinical disease [50]. A single-center retrospective study of 348 hypertensive adults found that an AI-enhanced ECG model accurately detected speckle-tracking GLS-defined subclinical LV dysfunction (LVEF ≥50% with GLS > −18%) with an area under the receiver-operating-characteristic curve of 0.86 (95% confidence interval (CI): 0.82-0.89), using speckle-tracking GLS as the reference standard; the AI-ECG probability score was independently associated with subclinical LV dysfunction after adjustment for LV mass index, E/e′, age, and duration of hypertension [51]. While these results are encouraging, they are preliminary and will need prospective validation in multi-center and multi-ethnic populations prior to recommending the use of the AI-ECG model for screening purposes. In CMR, AI-based segmentation and analysis have demonstrated excellent diagnostic accuracy for various cardiovascular diseases and greatly reduced analysis time [52]. Explainable-AI techniques are under development that could provide the necessary insights to encourage broader clinical use and regulatory support [53].

Screening Protocols and Future Research Priorities

Addressing these issues is essential to translate these capabilities and to promote widespread adoption of practical screening. Detailed cost-effectiveness analyses will be required to determine the appropriate populations for screening using STE and/or CMR techniques. Further development of T1-mapping sequences will be necessary to achieve robust normal ranges across different ethnic groups and field strengths [54], and native T1 in isolation is relatively non-specific and requires evaluation in conjunction with other clinical and imaging parameters. Larger multicenter prospective studies are warranted to examine the incremental value of the novel circulating biomarkers and imaging techniques for prediction and to evaluate whether therapeutic intervention guided by advanced imaging modalities leads to improved patient outcomes. Such studies should also be dedicated to defining the optimal sex- and ethnicity-specific cut-offs for GLS and ECV values.

Equity of access is also paramount. CMR is still relatively costly, time-consuming, and highly rationed in most countries. Considering that the majority of people with hypertension reside in low- and middle-income countries with limited availability of CMR [55], the immediate focus is to implement low-cost and high-volume screening methodologies. Other diagnostic tools with lower cost and higher portability, such as STE and AI-assisted ECG, may be more feasible as a primary screening tool in these countries, with CMR used as an adjunct for confirmation and phenotyping where available. The integration of clinical variables, biomarkers, imaging metrics, and genetic information through an AI-based platform may guide more precision-based treatment strategies for hypertension [56].

## Conclusions

A new paradigm of hypertensive cardiac damage is emerging. As we have shown in this review, myocardial remodeling in both borderline and established hypertension likely occurs much earlier than the development of LVH, as detected by conventional methods. Diffuse interstitial fibrosis, neurohormonal activation, microvascular dysfunction, and impaired myocardial strain represent early manifestations of hypertensive heart disease, undetectable by conventional ECG and echocardiography. STE, with the assessment of GLS, is a useful tool to detect subclinical systolic dysfunction, and CMR is excellent in tissue characterization (e.g., detection of diffuse fibrosis by T1 mapping and ECV). In sum, LVH should not be viewed as the earliest manifestation of hypertensive heart damage; rather, it often reflects a later structural stage preceded by cellular, interstitial, and functional abnormalities.

Earlier identification of subclinical remodeling allows intervention at a potentially modifiable stage, improved risk stratification facilitates more precise prevention strategies, and imaging monitoring of treatment response enables more personalized therapy. In conjunction with biomarkers and AI, advanced imaging techniques might help revolutionize hypertension management from a numbers-based approach to a complication-based approach focused on early detection and targeted prevention. We have the means to detect these early alterations; it is up to the medical community to use them to the full and equitable advantage of the population.

## References

[1] (2026). Hypertension. https://www.who.int/news-room/fact-sheets/detail/hypertension.

[2] (2021). Worldwide trends in hypertension prevalence and progress in treatment and control from 1990 to 2019: a pooled analysis of 1201 population-representative studies with 104 million participants. Lancet.

[3] Mills KT, Stefanescu A, He J (2020). The global epidemiology of hypertension. Nat Rev Nephrol.

[4] Levy D, Garrison RJ, Savage DD, Kannel WB, Castelli WP (1990). Prognostic implications of echocardiographically determined left ventricular mass in the Framingham Heart Study. N Engl J Med.

[5] Nwabuo CC, Vasan RS (2020). Pathophysiology of hypertensive heart disease: beyond left ventricular hypertrophy. Curr Hypertens Rep.

[6] González A, López B, Ravassa S, San José G, Latasa I, Butler J, Díez J (2024). Myocardial interstitial fibrosis in hypertensive heart disease: from mechanisms to clinical management. Hypertension.

[7] Whelton PK, Carey RM, Aronow WS (2018). 2017 ACC/AHA/AAPA/ABC/ACPM/AGS/APhA/ASH/ASPC/NMA/PCNA Guideline for the Prevention, Detection, Evaluation, and Management of High Blood Pressure in Adults: a report of the American College of Cardiology/American Heart Association Task Force on Clinical Practice Guidelines. Hypertension.

[8] Williams B, Mancia G, Spiering W (2018). 2018 ESC/ESH Guidelines for the management of arterial hypertension. Eur Heart J.

[9] Mancia G, Kreutz R, Brunström M (2023). 2023 ESH Guidelines for the management of arterial hypertension: The Task Force for the management of arterial hypertension of the European Society of Hypertension: endorsed by the International Society of Hypertension (ISH) and the European Renal Association (ERA). J Hypertens.

[10] McEvoy JW, McCarthy CP, Bruno RM (2024). 2024 ESC Guidelines for the management of elevated blood pressure and hypertension. Eur Heart J.

[11] Huang Y, Wang S, Cai X, Mai W, Hu Y, Tang H, Xu D (2013). Prehypertension and incidence of cardiovascular disease: a meta-analysis. BMC Med.

[12] Baethge C, Goldbeck-Wood S, Mertens S (2019). SANRA-a scale for the quality assessment of narrative review articles. Res Integr Peer Rev.

[13] Zdravkovic M, Klasnja S, Popovic M (2022). Cardiac magnetic resonance in hypertensive heart disease: time for a new chapter. Diagnostics (Basel).

[14] Treibel TA, Zemrak F, Sado DM (2015). Extracellular volume quantification in isolated hypertension - changes at the detectable limits?. J Cardiovasc Magn Reson.

[15] Huang X, Hu L, Long Z, Wang X, Wu J, Cai J (2024). Hypertensive heart disease: mechanisms, diagnosis and treatment. Rev Cardiovasc Med.

[16] Clarke C, Flores-Muñoz M, McKinney CA, Milligan G, Nicklin SA (2013). Regulation of cardiovascular remodeling by the counter-regulatory axis of the renin-angiotensin system. Future Cardiol.

[17] Paz Ocaranza M, Riquelme JA, García L, Jalil JE, Chiong M, Santos RA, Lavandero S (2020). Counter-regulatory renin-angiotensin system in cardiovascular disease. Nat Rev Cardiol.

[18] Ávila-Martínez DV, Mixtega-Ruiz WK, Hurtado-Capetillo JM, Lopez-Franco O, Flores-Muñoz M (2024). Counter-regulatory RAS peptides: new therapy targets for inflammation and fibrotic diseases?. Front Pharmacol.

[19] González A, Ravassa S, López B (2018). Myocardial remodeling in hypertension. Hypertension.

[20] Ikonomidis I, Tzortzis S, Paraskevaidis I (2012). Association of abnormal coronary microcirculatory function with impaired response of longitudinal left ventricular function during adenosine stress echocardiography in untreated hypertensive patients. Eur Heart J Cardiovasc Imaging.

[21] Ismail TF, Frey S, Kaufmann BA, Winkel DJ, Boll DT, Zellweger MJ, Haaf P (2023). Hypertensive heart disease - the imaging perspective. J Clin Med.

[22] Lip GY, Coca A, Kahan T (2017). Hypertension and cardiac arrhythmias: a consensus document from the European Heart Rhythm Association (EHRA) and ESC Council on Hypertension, endorsed by the Heart Rhythm Society (HRS), Asia-Pacific Heart Rhythm Society (APHRS) and Sociedad Latinoamericana de Estimulación Cardíaca y Electrofisiología (SOLEACE). Europace.

[23] Nemtsova V, Burkard T, Vischer AS (2023). Hypertensive heart disease: a narrative review series - part 2: macrostructural and functional abnormalities. J Clin Med.

[24] Chen J, Yang X, Li X (2024). Association between myocardial layer-specific strain and high 10-year risk of atherosclerotic cardiovascular disease in hypertension-findings from the China-PAR project study. Front Cardiovasc Med.

[25] Smiseth OA, Torp H, Opdahl A, Haugaa KH, Urheim S (2016). Myocardial strain imaging: how useful is it in clinical decision making?. Eur Heart J.

[26] Levy D, Labib SB, Anderson KM, Christiansen JC, Kannel WB, Castelli WP (1990). Determinants of sensitivity and specificity of electrocardiographic criteria for left ventricular hypertrophy. Circulation.

[27] Jain A, Tandri H, Dalal D (2010). Diagnostic and prognostic utility of electrocardiography for left ventricular hypertrophy defined by magnetic resonance imaging in relationship to ethnicity: the Multi-Ethnic Study of Atherosclerosis (MESA). Am Heart J.

[28] Peguero JG, Lo Presti S, Perez J, Issa O, Brenes JC, Tolentino A (2017). Electrocardiographic criteria for the diagnosis of left ventricular hypertrophy. J Am Coll Cardiol.

[29] Grothues F, Smith GC, Moon JC (2002). Comparison of interstudy reproducibility of cardiovascular magnetic resonance with two-dimensional echocardiography in normal subjects and in patients with heart failure or left ventricular hypertrophy. Am J Cardiol.

[30] Armstrong AC, Gjesdal O, Almeida A (2014). Left ventricular mass and hypertrophy by echocardiography and cardiac magnetic resonance: the multi-ethnic study of atherosclerosis. Echocardiography.

[31] Voigt JU, Pedrizzetti G, Lysyansky P (2015). Definitions for a common standard for 2D speckle tracking echocardiography: consensus document of the EACVI/ASE/Industry Task Force to standardize deformation imaging. Eur Heart J Cardiovasc Imaging.

[32] Farsalinos KE, Daraban AM, Ünlü S, Thomas JD, Badano LP, Voigt JU (2015). Head-to-head comparison of global longitudinal strain measurements among nine different vendors: the EACVI/ASE Inter-Vendor Comparison Study. J Am Soc Echocardiogr.

[33] Rodrigues JC, Amadu AM, Dastidar AG (2016). Comprehensive characterisation of hypertensive heart disease left ventricular phenotypes. Heart.

[34] Hamilton-Craig CR, Strudwick MW, Galloway GJ (2016). T1 mapping for myocardial fibrosis by cardiac magnetic resonance relaxometry - a comprehensive technical review. Front Cardiovasc Med.

[35] Moon JC, Messroghli DR, Kellman P (2013). Myocardial T1 mapping and extracellular volume quantification: a Society for Cardiovascular Magnetic Resonance (SCMR) and CMR Working Group of the European Society of Cardiology consensus statement. J Cardiovasc Magn Reson.

[36] Noori AA, Barzani MA (2022). Predictors of impaired left ventricular global longitudinal strain in patients with essential hypertension and preserved ejection fraction. Open Cardiovasc Med J.

[37] Oh JK, Park JH (2022). Role of strain echocardiography in patients with hypertension. Clin Hypertens.

[38] Tadic M, Gherbesi E, Sala C, Carugo S, Cuspidi C (2022). Effect of long-term antihypertensive therapy on myocardial strain: a meta-analysis. J Hypertens.

[39] Biering-Sørensen T, Biering-Sørensen SR, Olsen FJ (2017). Global longitudinal strain by echocardiography predicts long-term risk of cardiovascular morbidity and mortality in a low-risk general population: the Copenhagen City Heart Study. Circ Cardiovasc Imaging.

[40] Kadoglou NP, Mouzarou A, Hadjigeorgiou N, Korakianitis I, Myrianthefs MM (2024). Challenges in echocardiography for the diagnosis and prognosis of non-ischemic hypertensive heart disease. J Clin Med.

[41] Wang S, Hu H, Lu M (2017). Myocardial extracellular volume fraction quantified by cardiovascular magnetic resonance is increased in hypertension and associated with left ventricular remodeling. Eur Radiol.

[42] Zhuang B, Cui C, Sirajuddin A (2020). Detection of myocardial fibrosis and left ventricular dysfunction with cardiac MRI in a hypertensive swine model. Radiol Cardiothorac Imaging.

[43] Kim MY, Cho SJ, Kim HJ, Kim SM, Lee SC, Paek M, Choe YH (2022). T1 values and extracellular volume fraction in asymptomatic subjects: variations in left ventricular segments and correlation with cardiovascular risk factors. Sci Rep.

[44] Doyle RS, Walsh R, Walsh J, Temperley HC, McCormick J, Giblin G (2025). Systematic review and meta-analysis of cardiac MRI T1 and ECV measurements in pre-heart failure populations. Hearts.

[45] Cauwenberghs N, Hedman K, Kobayashi Y, Vanassche T, Haddad F, Kuznetsova T (2019). The 2013 ACC/AHA risk score and subclinical cardiac remodeling and dysfunction: complementary in cardiovascular disease prediction. Int J Cardiol.

[46] Ojji D, Libhaber E, Lamont K, Thienemann F, Sliwa K (2020). Circulating biomarkers in the early detection of hypertensive heart disease: usefulness in the developing world. Cardiovasc Diagn Ther.

[47] Mbeta E, Williams K, Yates J, Sankaranarayanan R, Penson P, Lip GY, McDowell G (2025). Evaluating the prognostic significance of circulating biomarkers of end organ damage in hypertension. J Clin Med.

[48] Cai A, Bayes-Genis A, Parati G (2026). Natriuretic peptides and cardiac troponins in hypertension and heart failure: a contemporary review of the evidence. J Hum Hypertens.

[49] Litjens G, Ciompi F, Wolterink JM, de Vos BD, Leiner T, Teuwen J, Išgum I (2019). State-of-the-art deep learning in cardiovascular image analysis. JACC Cardiovasc Imaging.

[50] Sehly A, Jaltotage B, He A (2022). Artificial intelligence in echocardiography: the time is now. Rev Cardiovasc Med.

[51] Bayraktar MF (2026). Artificial intelligence-enhanced electrocardiography for identifying subclinical left ventricular dysfunction in hypertensive individuals: a comprehensive clinical evaluation. Front Cardiovasc Med.

[52] Wang YJ, Yang K, Wen Y (2024). Screening and diagnosis of cardiovascular disease using artificial intelligence-enabled cardiac magnetic resonance imaging. Nat Med.

[53] Salih A, Boscolo Galazzo I, Gkontra P, Lee AM, Lekadir K, Raisi-Estabragh Z, Petersen SE (2023). Explainable artificial intelligence and cardiac imaging: toward more interpretable models. Circ Cardiovasc Imaging.

[54] Messroghli DR, Moon JC, Ferreira VM (2017). Clinical recommendations for cardiovascular magnetic resonance mapping of T1, T2, T2* and extracellular volume: a consensus statement by the Society for Cardiovascular Magnetic Resonance (SCMR) endorsed by the European Association for Cardiovascular Imaging (EACVI). J Cardiovasc Magn Reson.

[55] Kario K, Okura A, Hoshide S, Mogi M (2024). The WHO Global report 2023 on hypertension warning the emerging hypertension burden in globe and its treatment strategy. Hypertens Res.

[56] Sengupta PP, Shrestha S (2019). Machine learning for data-driven discovery: the rise and relevance. JACC Cardiovasc Imaging.

